# Prospects of IL-2 in Cancer Immunotherapy

**DOI:** 10.1155/2018/9056173

**Published:** 2018-05-06

**Authors:** Hani Choudhry, Nawal Helmi, Wesam H. Abdulaal, Mustafa Zeyadi, Mazin A. Zamzami, Wei Wu, Maged Mostafa Mahmoud, Mohiuddin Khan Warsi, Mahmood Rasool, Mohammad S. Jamal

**Affiliations:** ^1^Department of Biochemistry, Cancer Metabolism and Epigenetic Unit, Faculty of Science, Cancer and Mutagenesis Unit, King Fahd Center for Medical Research, King Abdulaziz University, Jeddah, Saudi Arabia; ^2^Department of Medicine, University of California, San Francisco, CA 94143, USA; ^3^King Fahd Medical Research, King Abdulaziz University, Jeddah, Saudi Arabia; ^4^Department of Molecular Genetics and Enzymology, Division of Human Genetics and Genome Research, National Research Center, Giza, Egypt; ^5^Department of Biochemistry, University of Jeddah, Jeddah, Saudi Arabia; ^6^Center of Excellence in Genomic Medicine Research, King Abdulaziz University, Jeddah, Saudi Arabia

## Abstract

IL-2 is a powerful immune growth factor and it plays important role in sustaining T cell response. The potential of IL-2 in expanding T cells without loss of functionality has led to its early use in cancer immunotherapy. IL-2 has been reported to induce complete and durable regressions in cancer patients but immune related adverse effects have been reported (irAE). The present review discusses the prospects of IL-2 in immunotherapy for cancer.

## 1. Introduction

Interleukin-2 (IL-2) was identified in the supernatants of activated T cells over three decades ago [1, 2]. IL-2 is the first cytokine for which receptor component was cloned [3, 4]. IL-2 is a four *α*-helical bundle cytokine of 15.5 kDa size. It is mainly produced by CD4+ T cells as a result of antigen stimulation response [5]. However, to some extent, IL-2 is also produced by NK T cells [6], CD8+ cells [7], mast cells [8], and dendritic cells (DCs) [9]. IL-2 possesses potent T cell growth factor action. It can also induce natural killer (NK) cells and potentiate their cytolytic effect and promote many other immune system components which are required for the removal of autoreactive cells and maintenance of homeostasis [10]. In the last two decades, the potential of IL-2 to expand T cells without affecting its activity has led to identification of its potential as an immunotherapeutic agent against cancer. The IL-2 administration is reported to induce apparently curative and durable regressions in cancer patients. In 1988, UFDA approved IL-2 for therapy of metastatic melanoma and then in 1992 for renal cell cancer. Further research also led to evolution of cell transfer therapies having promising response against melanoma cases [11]. This review discusses scope of IL-2 in the immunotherapy of cancer and major challenge in the development of IL-2 based therapeutic approach as well as perspective on future research.

## 2. IL-2 Receptor and Signaling

IL-2 is a T cell-derived common cytokine. It plays vital role in growth as well as differentiation of T cells, B cells, natural killer cells, and many other cell types [12]. The signaling pathway of IL-2 is mediated by a selective receptor family [13], which includes three classes of cell surface receptors: the alpha (*α*), beta (*β*), and gamma (*γ*) chains. The dimeric low affinity IL-2 receptor consists of the *γ* and *β* chains and is expressed on T cells and NK cells. The high affinity IL-2 receptor consisting of the *α*, *β*, and *γ* chains is expressed on Tregs and activated T cells [14, 15]. The activated lymphocytes also express these high affinity receptors. The low and high affinity IL-2 receptors are expressed in steady state. The transcription of IL-2R is induced by various factors. Transcription of IL-2R is induced on T cells which are activated by TCR or IL-2 on T cells. Transcription of IL-2R is induced by various factors. Transcription of IL-2R*α* is induced on T cells which are activated by TCR or IL-2 [16]. IL-2R*α* transcription is also induced by intermediate affinity receptors upon binding of IL-2 and as a response to T cell activation. There is also rapid formation of high affinity receptors and consequent increase in responsiveness to IL-2.

Expression of IL-2R*β* chain is also induced by IL-2 on T cells [23]. These cells also have *γ* chain expression but it is less inducible than IL-2R*α* or IL-2R*β* [24]. IL-2R*α* is also expressed by NK cells, B cells, mature dendritic cells (DCs), and endothelial cells [25–28]. This binding also promotes cytolytic activity and cell growth [29].

IL-2 presented in trans and bound to cellular IL-2R*α* can also activate another cell having IL-2R*β* and *γ* chain expression [30]. However, it should be noted that the affinity with which IL-2 can bind to IL-2R*α* is relatively low with rapid on and off rates.

**Figure 1:**
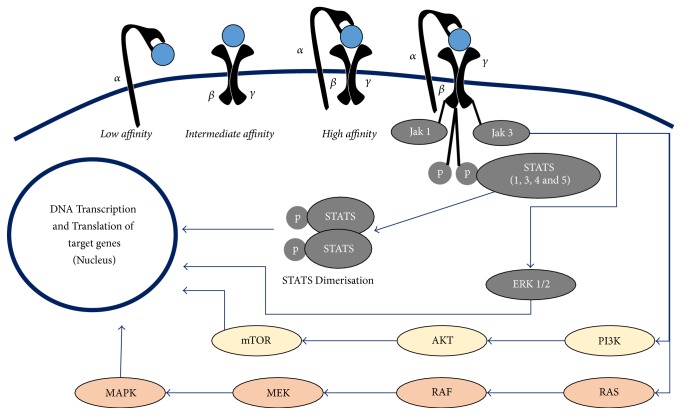
Showing three different types of IL-2 receptors.

IL-2 binding to IL-2R*αβγ* or IL-2R*βγ* complex initiates signal transduction for the transcription of target genes through multiple signaling pathways. These include Janus kinase (JAK) signal transducer and activator of transcription (STAT) pathway, the phosphoinositide 3-kinase (PI3K) AKT pathway, and the mitogen-activated protein kinase (MAPK) pathway (Figure 1). All of these three major pathways mediate the effect of IL-2 on cell proliferation, activation, differentiation, survival, and cytokine production in the immune cells [31, 32].

## 3. Cancer Immunotherapy Using IL-2

It has been long established that the immune system can be harnessed against neoplastic cells. However, IL-2 was the first cytokines to be successfully used in the treatment of cancer. This was because it can promote T cells as well as NK cells. IL-2 can induce T cell proliferation and differentiation and also cause its activation. The complexing IL-2 with anti-IL-2 mAbs has ability to potentiate signaling via the intermediate affinity CD122/CD132 receptor in vivo. Kamimura and Bevan examined the effect of treatment of naive CD8+ T cells with IL-2 signals in vivo. Extensive division was observed in T cell upon treatment of the host animals with IL-2 and anti-IL-2 complexes in the absence of any other stimulation. The potent IL-2 signals caused proliferation and differentiation of naive CD8+ T cells into functional memory cells having conventional central memory phenotype [33].

Further, lymphokine activated killer (LAK) cells represent a unique and fundamental cytotoxic effector system plays a role in immune surveillance against NK resistant solid tumor cells and has role in the adoptive immunotherapy. LAK cells are a heterogeneous mixture of ex vivo expanded and activated T, NK, and NKT cells which display major histocompatibility complex (MHC) nonrestricted cytotoxicity that do not rely on HLA-mediated recognition of tumor targets. LAK cells can recognize and kill human cancer cells as well as cultured tumor cell lines without any need for further* in vitro* stimulation [34].* In vitro* grown LAK cells have also demonstrated* in vivo *activity against tumor-bearing mice. However, in all these studies, the tumors were treated before vascularization [35]. Further, clinical trials using LAK cells and recombinant LAK cells conducted in 30 patients with advanced cancer did not produce any antitumor response [36]. Later, studies in murine models showed that* in vivo* activity of LAK cells was increased by administration of IL-2. Based on this observation, the attempts were made to administer LAK cells in combination with maximum tolerated dose of IL-2 in humans. Recombinant interleukin-2 (IL-2) therapy was first tried in 1984 and its novel effects in regulating regulatory T cells apart from effector T cells were identified after its FDA approval [37]. In one of the first studies, in case of metastatic renal cell cancer, ten patients (7%) exhibited complete regression whereas partial regression was observed in twenty (approximately 13%). In case of metastatic melanoma, nine patients (7%) achieved complete regression. Complete remission was seen in 15 cases for seven months to as long as ninety-one months [17]. Further, the therapy showed durable effect with ongoing complete responses over a duration of 39 to 148 months [38]. A few other clinical studies have also demonstrated safety of infusing autologous leukocytes in high-grade glioma patients with local injection of LAK cells. Outcomes of the studies in terms of prolonging disease free survival are also promising. However, comparison of therapy of IL-2 alone with combination of IL-2 with LAK cell has not shown any significant difference in response to renal cell carcinoma in clinical setting [39, 40].

The durability of the response which occurs due to development of T cell memory appears to be most important feature of cancer immunotherapy.

Recent studies showed that immunization with combination of IL-2 and an altered peptide ligand derived from gp100 (melanoma associated protein) has better overall clinical response. In this case, the progression-free survival was of relatively longer duration than IL-2 treatment alone. This observation was in line with the idea that IL-2 can not only maintain but also enhance the tumor reactive T cells. However, as evident from the results, the treatment was effective on fraction of patients [19]. More recently, better IL-2 based cell transfer therapies with effective response in melanoma have been developed. Further, genetic modification of T cells with *αβ* TCRs or chimeric Ag receptors encoding gene and administration of these cells after expansion in IL-2 have opened the scope for use of cell transfer therapy in other cancer types [11].

The immunotherapy approach causes development of lifelong immunologic memory and consequent durable response. However, the questions that need to be addressed include the evaluation of survival benefits and the scope for possible retreatment. To answer these questions, much larger clinical trials with extended follow-up period should be conducted.

Outcome of trials using combination of IL-2 and interferon have found it to be nonsuperior to high dose IL-2 as a single agent [41–43]. Large trials on biochemotherapy involving combination of chemotherapeutic agent with IL-2 several large trials have consistently shown better overall response rates with early clinical benefit as compared with chemotherapy [44–47]. However, early response and benefit do not affect overall survival due to short duration of response [48, 49]. Maintenance biotherapy after induction of biochemotherapy has been tried to extend the response, but with limited success [50]. The exact mechanism of toxicity due to combination of IL-2 with interferon or chemotherapeutic agents is complex and yet to be fully understood [51]. Patients sometime require interruption or discontinuation because of toxicity [17, 52]. The toxicities due to treatment with IL-2 alone are both predictable and manageable. Further, toxicity in this case tapers off quickly following therapy [48, 53]. In spite of this, biochemotherapy remains an option in cases of disease with rapid progression.

## 4. Dual Effect of IL-2: Major Challenge in the Development of Promising Immunotherapy

The major challenge in the development of IL-2 as a therapeutic antitumor agent is that IL-2 can act on both T cells and Tregs. Thus, reports on use of IL-2 have used two different strategies, one to reduce the autoimmune responses and another to augment immune responses against tumor (Table 1). Recently, studies have used IL-2 in low dose, either alone or in combination, to induce preferential activation of Tregs. Tregs having high affinity for IL-2 can compete more effectively for it at low IL-2 levels [54]. Few studies involving HCV induced vasculitis and Graft-Versus-Host Disease showed improvement in clinical outcome based on the described concept of IL-2 therapy using low doses. However, in the study involving renal cancer and melanoma, subjects which were given high dose of IL-2 showed limited efficacy due to increased Treg level [18, 22, 55]. Development of somewhat lethal toxicity is another major limitation of therapy with IL-2 at high doses. Also, IL-2 has a very short life in systemic delivery. Attempts made to reduce the side effects by lowering dose resulted in marked loss of therapeutic effect due to dominant effect of immunosuppressive Treg cell leading to poor outcomes in cancer patients [18]. Further studies are needed to explore the dual effect of IL-2 in cancer therapy. The translation of effect of IL-2 on Tregs and effector cell from preclinical to clinical setting is not always predictable [56, 57]. This is so because there are a number of factors influencing the outcome in clinical condition (e.g., genetic variability, disease factor, and most importantly the variation in Tregs and effector cells immune response in individuals). There is also an unmet need for better biomarkers which can be used to predict the response of IL-2 immunotherapy and hence can predict factors like genetic polymorphisms or serum proteins or antigen expression. This can also lead to development of a more personalized therapy approach based on the individual characteristics of patient especially the ones who are expected to benefit from IL-2 therapy.

## 5. Future Perspective of IL-2 Based Immunotherapy

IL-2 was one of the first cytokines exploited for development of tumor immunotherapy. However, the contrasting action of IL-2 has led to confusing response and limited the development of IL-2 for tumor immunotherapy. Further, the delivery of IL-2 has been associated with multiple side effects, further affecting its utility in clinical setting. Development of IL-15 for cancer immunotherapy was initiated in the last decade and it was viewed as a safer successor to IL-2-based immunotherapies since both cytokines have shared receptor subunits and activated similar downstream pathways. Preclinical data suggested IL-15 to be safer alternative to IL-2 immunotherapy [58]. However, translational challenges and issues in delivery affected its clinical development [58]. However, recently Nektar Therapeutics has developed a memory T cell stimulating cytokine, NKTR255, which causes long-term T cell activation through IL-15 pathway. According to the company, it has been found to improve the quality of T cell memory response to treat cancer. Through optimal engagement of the, NKTR-255 stimulates proliferation and survival of CD8+ T cells and natural killer (NK) cells through optimal engagement of IL-15R*α*/IL-2R*γ* receptor complex and induces long-term immunological memory for sustained antitumor immune response [59]. Recently a complex between a novel human IL-15 superagonist variant and a human IL-15R*α* sushi domain-Fc fusion protein, termed ALT-803, has been created. ALT-803 showed promising immunostimulatory activity and antimyeloma activity in mouse model with relatively longer half-life than IL-15 and wide therapeutic dose range. The potent immunostimulatory activity of ALT-803 is also attributed to its prolonged retention in lymphoid organs compared to IL-15 [60, 61].

Development of better understanding of immune responses of IL-2 can open new windows for developing better immunotherapy. Many strategies are being developed to improve efficacy, while reducing the toxicity of IL-2 therapy. The recent evidence suggests that genetic variation plays an important role in defining the clinical output of immunotherapy. Some studies have identified functional polymorphism as a reason for poor response to therapies [62, 63]. Further exploration of polymorphism in IL-2 gene can help in elucidation of clinical response. Efforts have also been focused on development of mutant form of IL-2 with preferential binding to different IL-2R. This has potential to bypass interaction of IL-2 with other subtypes of receptors [64]. This strategy has been tried as part of Phase I study against cases of renal cancer and advanced melanoma [65]. Mutational approaches to construct IL-2 mutants with varying binding affinities for components of the IL-2 receptor have been tried in few reports. This includes strategy to enhance binding to IL-2R*α* chain. The objective is to improve IL-2 efficacy based on the fact that effect T cells have relatively higher expression of *α* chain. On the contrary, alternate strategy is to employ creation of mutants having impaired binding to *β* chain. This can help in reducing expression of NK cells which have higher *βγ* expression while retaining the property for effector T cell stimulation [66–69]. One study used IL-2 mutants having enhanced binding to *β* chain. The residues in the mutant IL-2 caused conformation effect in IL-2 leading to improved binding to the *β* chain. One of the mutants is known as “superkine” due to its enhanced agonistic effect. This superkine also exhibited relatively improved antitumor activity with relatively lesser side effects compared to systemic IL-2 therapy [70]. Improvement of half-life of IL-2 is another important aspect for improving development of effective IL-2 based immunotherapy. This can greatly reduce the dose of IL-2, while leaving a positive shift in clinical outcome [71]. Combination of IL-2 with monoclonal antibodies is also being utilized as a new approach in cancer immunotherapy. It can be used for targeted approach against specific cells based on the affinity of their IL-2R. The immunocytokines, namely, antibody-cytokine fusion proteins, have already been tested in preclinical cancer models [29]. Sockolosky et al. have recently developed engineered IL-2 cytokine-receptor orthogonal (ortho) pairs that interact with one another. This ortho IL-2 transmits native IL-2 signals but does not interact with their natural cytokine and receptor counterparts since it lacks detectable binding to IL-2. Ortho IL-2 pairs showed good efficacy as in mouse model of adoptive cell therapy [72].

IL2 immunotherapy, being effective treatment option, is associated with various toxicities. Developing predictive biomarker can help optimize selection of appropriate patient population which is likely to be most benefited from therapy. Recently, Kuzman et al. have shown the positive relation between Neutrophil-lymphocyte ratio and response to high dose interleukin-2 in patients with renal cell carcinoma [73]. Identification of more selective biomarkers for IL-2 immunotherapy against various cancer types can lead to extension of benefits of IL-2 immunotherapy in a cost effective manner while limiting its toxicity in larger population.

There are still a number of issues that should be addressed for successful translation of IL-2 therapy in clinical setting. These include optimization of dose of mutants and other IL-2 based regimens, the peripheral effects and immunogenicity of the new molecules. Future therapy will not only depend on IL-2 alone but a combination of measures including other immunologic options, like antibody treatments and active vaccinations. Determination of optimum combinations/approach using the massive research data being generated at a fast pace should yield greater clinical benefits in future.

## Figures and Tables

**Table 1:** Selected clinical studies with IL-2 treatment at different dose levels.

Treatment type	Disease condition	Treatment	Comments	Ref.
High dose treatment	Melanoma/renal cell cancer	720,000 IU/kg of i.v. IL-2 given eight hourly (up to 15 doses per cycle)	Complete response in 7% and partial regression in 10% of metastatic melanoma patients Complete regression in 7% and partial regression in 13% patients of renal cell carcinoma	[17]
	Metastatic melanoma/renal cell carcinoma	720,000 IU/kg of i.v. IL-2 given eight hourly (up to 15 doses per cycle)	The proportion of CD4+CD25hi T cells in total CD4 T cells showed 6-fold increase compared to pretreatment level	[18]
	Melanoma	IL-2 as high-dose bolus 8 hourly or gp100 single dose per cycle, along with high-dose IL-2 on the second day	The combination of interleukin-2 and gp100:209–217 (210M) peptide vaccine exhibited relatively higher response rate compared to interleukin-2 alone	[19]
	Renal cell carcinoma	720,000 or 600,000 IU/kg of i.v. IL-2 given 8 hourly to a maximum of 14 doses per cycle The majority of patients were also given a second cycle of HD IL-2 after an approximate rest of 9 days	HD IL-2 as sole front-line therapy, in the absence of added therapy exhibited extended clinical benefit	[20]

Low dose treatment	Graft versus host disease	Low s.c. dose (300,000; 1,000,000 or 3,000,000 IU/m^2^) of IL-2 for eight weeks	Twelve out of the twenty three evaluated patients exhibited good responses at multiple sites. The sustained clinical and Immunologic responses was observed in patients who received IL-2 for extended period	[21]
	HCV-induced vasculitis	1,500,000 IU/day of for 5 days, followed by 3 × 10^6^ IU per day of IL-2 for 5-day given at 3rd, 6th, and 9th week	Eight out of ten patients showed improvement in vasculitis. Low-dose interleukin-2 administration caused increased percentage of forkhead box P3 (FOXP3+), CD25^high^, CD4+, and Tregs	[22]
